# Analysis of the impact of nodular calcification on clinical outcome after drug-coated balloon angioplasty for femoropopliteal lesions

**DOI:** 10.1186/s42155-025-00583-6

**Published:** 2025-08-16

**Authors:** Shinsuke Mori, Mitsuyoshi Takahara, Tatsuya Nakama, Kazuki Tobita, Naoki Hayakawa, Yo Iwata, Kazunori Horie, Kenji Suzuki, Norihiro Kobayashi, Yoshiaki Ito

**Affiliations:** 1 Department of Cardiology, Saiseikai Yokohama City Eastern Hospital, 3-6-1 Shimosueyoshi, Tsurumi-Ku, Yokohama City Kanagawa, 230-0012 Japan; 2 Department of Laboratory Medicine, Osaka University Graduate School of Medicine, Suita-shi, Japan; 3 Department of Cardiology, Tokyo Bay Medical Centre, Urayasu-shi, Japan; 4 Department of Cardiology, Shonan Kamakura General Hospital, Kamakura-shi, Japan; 5 Department of Cardiovascular Medicine, Asahi General Hospital, Asahi-shi, Japan; 6 Department of Cardiology, Funabashi Municipal Medical Center, Funabashi-shi, Japan; 7 Department of Cardiovascular Medicine, Sendai Kousei Hospital, Sendai-shi, Japan; 8 Department of Cardiology, Tokyo Saiseikai Central Hospital, Minato-ku, Tokyo, Japan

**Keywords:** Drug-coated balloon, Endovascular therapy, Femoropopliteal lesion, Nodular calcification, Calcification

## Abstract

**Purpose:**

This study aimed to reveal the impact of nodular calcification (NC) on restenosis risk in patients undergoing femoropopliteal drug-coated balloon (DCB) angioplasty for symptomatic atherosclerotic peripheral artery disease.

**Methods:**

We retrospectively analyzed 568 patients who underwent endovascular therapy with DCB for de novo femoropopliteal lesions under intravascular ultrasound guidance between November 2017 and February 2021 at seven cardiovascular centers in Japan. Patients with lesions without calcification were excluded from the study. The patients were classified into two groups based on the presence or absence of NC: the NC [ +] group (n = 200) and the NC [-] group (n = 368). The main outcome was the primary patency at 3 years. Cox proportional hazards analysis was used to determine whether NC was an independent predictor of clinical outcomes.

**Results:**

The 3-year primary patency rates were significantly lower in the NC [ +] group than in the NC [-] group (53.8% vs. 65.8%, p = 0.001). After multivariate analysis, the presence of NC was independently associated with restenosis risk; the adjusted hazard ratio was 1.61 (95% confidence interval 1.15 to 2.26, p = 0.006).

**Conclusion:**

The NC is an independent predictor of restenosis in patients undergoing DCB angioplasty for femoropopliteal lesions. Patients with NC had a significantly lower primary patency, highlighting their negative impact on clinical outcomes. Further research is required to establish evidence-based strategies for managing calcified femoropopliteal lesions.

**Supplementary Information:**

The online version contains supplementary material available at 10.1186/s42155-025-00583-6.

## Introduction

Advancements in peripheral vascular therapy have expanded the applicability of endovascular treatments for complex femoropopliteal lesions. Endovascular therapy (EVT) has become the first-line treatment, replacing surgical options, owing to its high initial success rate and low risk of complications [1, 2].

Among endovascular modalities, drug-coated balloons (DCBs) have demonstrated superior outcomes compared to uncoated balloons, including higher primary patency rates and reduced risk of target lesion revascularization (TLR). Furthermore, DCBs have been confirmed to provide safety benefits over short- and long-term follow-up [3–5]. Notably, DCBs do not require stent implantation, allowing the preservation of arterial flexibility, reduced risk of restenosis, and greater flexibility for future treatment options. Therefore, they are often favored over stents for femoropopliteal lesions [6, 7]. However, calcified lesions remain a major challenge because the extent and severity of calcification are known predictors of restenosis following DCB therapy [8, 9].

Nodular calcification (NC), a distinct form of arterial calcification, has been identified as a significant risk factor for restenosis following percutaneous coronary intervention.

In the coronary arteries, NC is strongly associated with in-stent restenosis, primarily because of the recurrent protrusion of NC through stent struts after drug-eluting stent (DES) implantation [10].

Moreover, in patients with acute coronary syndrome (ACS), the presence of NC is associated with a significantly higher risk of ACS recurrence and TLRs. Continuous growth and re-protrusion of calcified lesions within the stent remain the primary causes of restenosis [11].

Despite these findings in the coronary arteries, there is a lack of sufficient data on the effect of NC on the femoropopliteal arteries, particularly after DCB angioplasty. This study aimed to evaluate the influence of NC on the restenosis risk in patients undergoing femoropopliteal DCB angioplasty for symptomatic atherosclerotic peripheral artery disease.

## Materials and methods

### Patients

The **ALLIGATOR** study (**A**nalysis of the Impact of nodu**L**ar ca**L**c**I**fication on Clinical Outcomes after dru**G**-coated balloon **A**ngioplas**T**y for fem**OR**opopliteal Lesions) retrospectively recruited patients who underwent EVT for Femoropopliteal Lesions between July 2017 and June 2020.

The inclusion criteria were as follows: (1) symptomatic peripheral artery disease (Rutherford categories 2–6), (2) treatment with DCB angioplasty, (3) treatment under intravascular ultrasound (IVUS) guidance, and (4) calcification detected by IVUS. In contrast, patients with (1) non-atherosclerotic disease, (2) life expectancy of less than 1 year, (3) acute limb ischemia, (4) non-calcified lesions, and (5) aneurysmal lesions were excluded. Thus, 568 patients were included in this study. These patients were classified into two groups based on the presence or absence of NC: NC [+] group (n = 200) and NC [-] group (n = 368).

The main outcome was the primary patency at 3 years. Cox proportional hazards analysis was used to determine whether NC was an independent predictor of clinical outcomes.

The study protocol was performed in accordance with the Declaration of Helsinki and approved by the local ethics committees of all participating institutions. The requirement for informed consent was waived due to the retrospective study design, in which existing medical records were used. Relevant information regarding the study is available to the public in accordance with the Ethical Guidelines for Medical and Health Research Involving Human Subjects.

### Procedure

The study utilized either a crossover or an ipsilateral approach by inserting a 4.5 to 7-Fr sheath into the common femoral artery. Guidewires ranging from 0.014 to 0.035 inches were employed during the procedure. After successful guidewire crossing, balloon angioplasty was performed using a conventional balloon. In cases of insufficient lesion dilatation, additional lesion preparation was aggressively performed using devices such as high-pressure balloons.

Following lesion dilatation with a balloon, DCBs, such as Lutonix (Becton and Dickinson Company, Franklin Lakes, New, USA) or IN.PACT Admiral (Medtronic Vascular, Santa Clara, CA, USA), were used when the blood vessels were adequately expanded without significant vessel dissection. The size of the DCB was selected based on the vessel diameter to cover the lesion fully, with the choice of DCBs depending on the operator. The IVUS images were assessed during the procedure by both the operator and an assisting physician to minimize interpretation bias. The patients were administered dual antiplatelet therapy with aspirin and thienopyridine for at least 1 month post-EVT. Atherectomy devices were not used during the study period because of their unavailability in our country.

### Definition

**Fig. 1:**
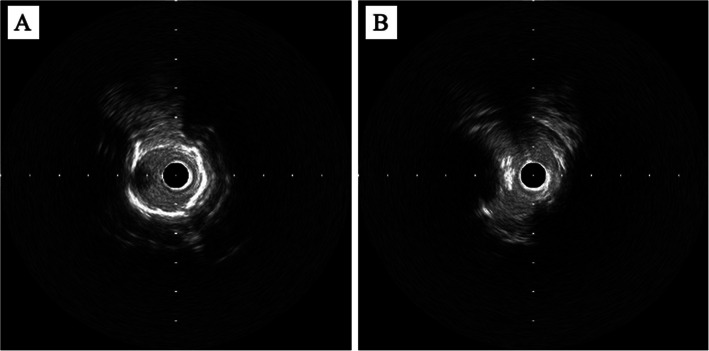
IVUS findings of calcification. **A** Superficial calcification, **B** Nodular calcification

Procedural success was defined as < 50% residual stenosis without severe vessel dissection. Primary patency was defined as the absence of restenosis and the need for revascularization of the treated lesion. Restenosis was defined as a peak systolic velocity ratio exceeding 2.4 on duplex ultrasound, more than 50% diameter stenosis, or occlusion during follow-up angiography or computed tomography. TLR refers to any subsequent revascularization required. Peripheral artery calcification scoring system (PACSS) classification was used to assess the degree of lesion calcification on angiography. It consisted of five grades based on calcification distribution and length: grade 0 (no visible calcification), grade 1 (unilateral wall calcification < 5 cm), grade 2 (unilateral calcification ≥ 5 cm), grade 3 (bilateral wall calcification < 5 cm), and grade 4 (bilateral calcification ≥ 5 cm) [12]. A nodular calcification appears as a distinct feature with an irregular, protruding, and convex luminal surface (Fig. 1) [13]. A poor run-off was characterized by either no infra-popliteal vessels or only one present.

### Statistical analysis

Data are presented as means ± standard deviation for continuous variables and as frequencies (percentages) for discrete variables unless otherwise indicated. Statistical significance was defined as a two-sided *P*-value of < 0.05, and 95% confidence intervals (CIs) were reported where appropriate. Intergroup differences in baseline characteristics were tested using Welch’s *t* test for continuous variables and the chi-square test for discrete variables. The association between baseline characteristics, except procedural features, and the presence of nodular calcification was investigated using a logistic regression model. The rates of freedom from restenosis, TLR, and limb salvage were estimated using the Kaplan–Meier method, and intergroup differences were tested using the log-rank test. Missing data were addressed using multiple imputations using the chained-equations method. We generated five imputed datasets and combined the analytical results based on Rubin’s rule. The interaction effect of chronic limb-threatening ischemia (CLTI) (versus intermittent claudication) on the association between the presence of nodular calcification and restenosis risk was also investigated using the Cox proportional hazards regression model. All statistical analyses were performed using R version 4.1.1 (R Development Core Team, Vienna, Austria).

## Result

The baseline characteristics of the study population are summarized in Table 1. There were no significant differences between the NC [+] and NC [-] groups in terms of age, sex, smoking status, diabetes mellitus, or chronic limb-threatening ischemia. A higher proportion of patients in the NC [+] group were on dialysis because of renal failure (43.5% vs. 35.1%; p = 0.059). However, this difference was not statistically significant. Reference vessel diameter was larger in the NC [+] group (31.5% vs. 21.5%, p = 0.015). Additionally, chronic total occlusion was more common in the NC [-] group (31.5% vs. 21.5%, p = 0.015). According to the PACSS classification, the NC [+] group was more likely to have severe calcifications (grade 4,55.5% vs. 20.9%, respectively). Similarly, the calcification arc was significantly larger in the NC [+] lesions (271–360° arc, 55.5% vs. 22.3%). Moreover, subintimal wire passage was significantly more frequent in the NC [+] group than in the NC [-] group (26.1% vs. 14.7%, p = 0.001). The IN.PACT Admiral was more commonly used in the NC [+] group (73.0% vs. 63.9%, p = 0.034). The minimum lumen area achieved after the procedure was similar between the NC [+] and NC [-] groups (14.0 ± 4.4 mm^2^ vs. 13.9 ± 5.6 mm^2^, p = 0.83).

**Table 1:** Baseline characteristics of the study population

	Overall population	Patients With NC	Patients without NC	P value
	(n = 568)	(n = 200)	(n = 368)	
Patient characteristics				
Male sex	388 (68.3%)	142 (71.0%)	246 (66.8%)	P = 0.36
Age (years)	74 ± 9	73 ± 9	74 ± 9	P = 0.24
Current smoking	117 (20.6%)	35 (17.5%)	82 (22.3%)	P = 0.22
Diabetes mellitus	370 (65.1%)	140 (70.0%)	230 (62.5%)	P = 0.089
Renal failure on dialysis	216 (38.0%)	87 (43.5%)	129 (35.1%)	P = 0.059
Limb characteristics				
Chronic limb-threatening ischemia	167 (29.4%)	59 (29.5%)	108 (29.3%)	P > 0.99
History of revascularization	291 (51.2%)	104 (52.0%)	187 (50.8%)	P = 0.86
Presence of popliteal lesion	275 (48.4%)	94 (47.0%)	181 (49.2%)	P = 0.68
Reference vessel diameter (mm)	5.2 ± 0.8	5.3 ± 0.7	5.1 ± 0.8	P = 0.005
Lesion length (mm)	153 ± 95	143 ± 91	158 ± 97	P = 0.061
Chronic total occlusion	159 (28.0%)	43 (21.5%)	116 (31.5%)	P = 0.015
PACSS classification				P < 0.001
Grade 0	127 (22.4%)	21 (10.5%)	106 (28.8%)	
Grade 1	98 (17.3%)	20 (10.0%)	78 (21.2%)	
Grade 2	49 (8.6%)	11 (5.5%)	38 (10.3%)	
Grade 3	106 (18.7%)	37 (18.5%)	69 (18.8%)	
Grade 4	188 (33.1%)	111 (55.5%)	77 (20.9%)	
IVUS findings				
Calcification arc				P < 0.001
1.(1–90)	177 (31.2%)	6 (3.0%)	171 (46.5%)	
2.(91–180)	118 (20.8%)	36 (18.0%)	82 (22.3%)	
3.(181–270)	80 (14.1%)	47 (23.5%)	33 (9.0%)	
4.(271–360)	193 (34.0%)	111 (55.5%)	82 (22.3%)	
Distal EEM area (mm2)	33.2 ± 11.7	33.3 ± 11.2	33.1 ± 12.0	P = 0.88
(missing data)	25 (4.4%)	6 (3.0%)	19 (5.2%)	P = 0.32
Procedural features				
Subintimal wire passage	106 (18.7%)	52 (26.1%)	54 (14.7%)	P = 0.001
(missing data)	2 (0.4%)	1 (0.5%)	1 (0.3%)	P > 0.99
IN.PACT Admiral use	381 (67.1%)	146 (73.0%)	235 (63.9%)	P = 0.034
Minimum lumen area (mm2)	14.0 ± 5.2	14.0 ± 4.4	13.9 ± 5.6	P = 0.83
(missing data)	92 (16.2%)	25 (12.5%)	67 (18.2%)	P = 0.10

*NC* nodular calcification, *PACSS* peripheral artery calcification scoring system, *IVUS* intravascular ultrasound, *EEM* external elastic membrane

**Fig. 2:**
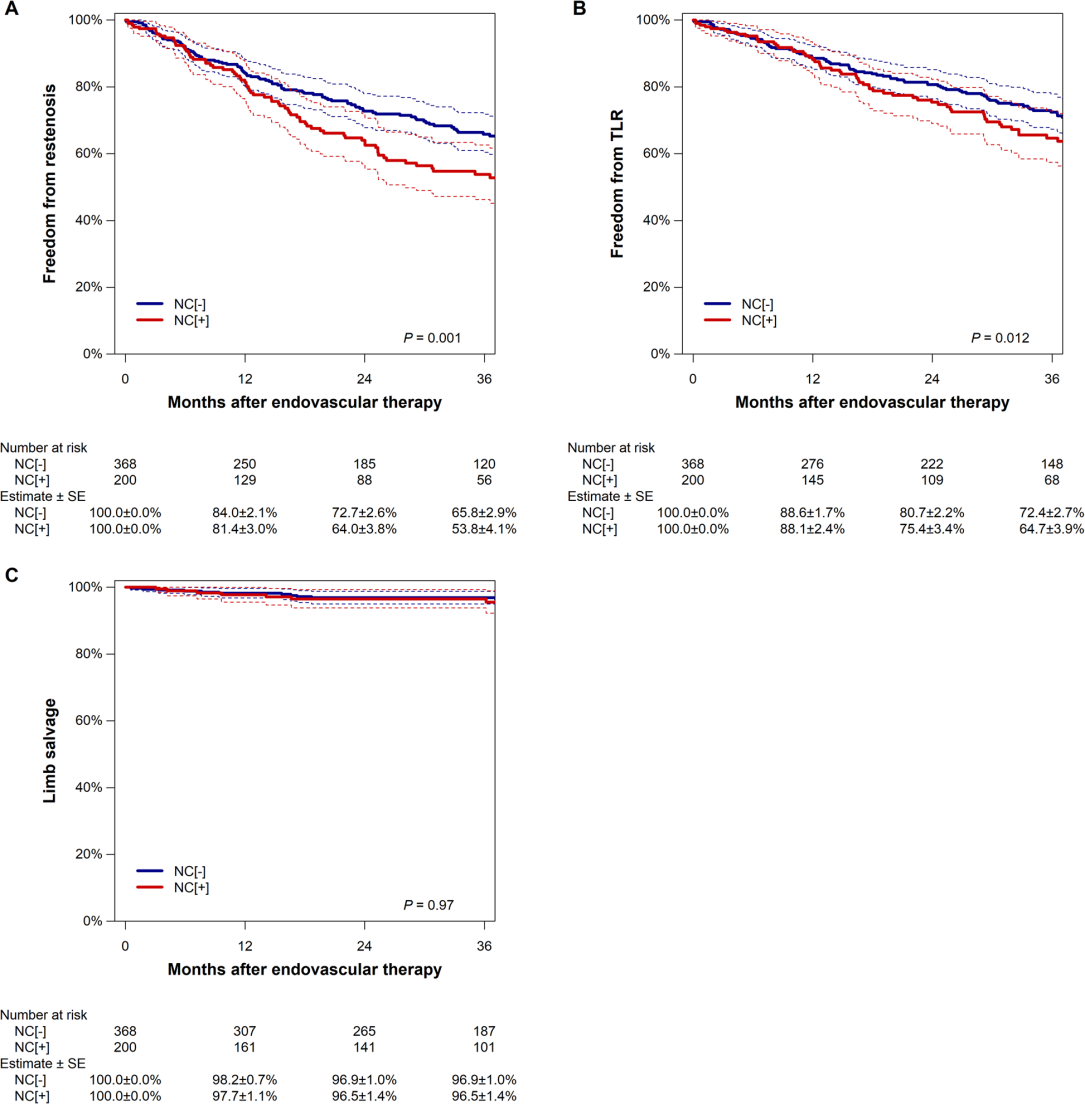
Kaplan–Meier estimates of freedom from restenosis (**A**), TLR (**B**), and limb salvage (**C**). The dotted lines indicate the 95% confidence intervals. NC, nodular calcification; SE, standard error; TLR, target lesion revascularization

During a median follow-up of 22.8 months (interquartile range, 7.0–38.4 months), 201 restenosis events were observed. Figure 2 illustrates the Kaplan–Meier estimates of freedom from restenosis and TLR. Patients with nodular calcification had a lower rate of freedom from restenosis (53.8% [95% CI, 46.3% to 62.6%] versus 65.8% [60.4% to 71.8%] at 36 months; P = 0.001) and TLR (64.7% [57.4% to 72.9%] versus 72.4% [67.4% to 77.8%] at 36 months; P = 0.012). On the other hand, the rate of limb salvage was similar between the two groups (96.5% [93.8% to 99.3%] versus 96.5% [95.0% to 98.8%] at 36 months; P = 0.97). In the subgroup of patients with CLTI, no statistically significant differences in clinical outcomes were observed between the NC [+] and NC [-] groups (Supplemental Figure).

**Fig. 3:**
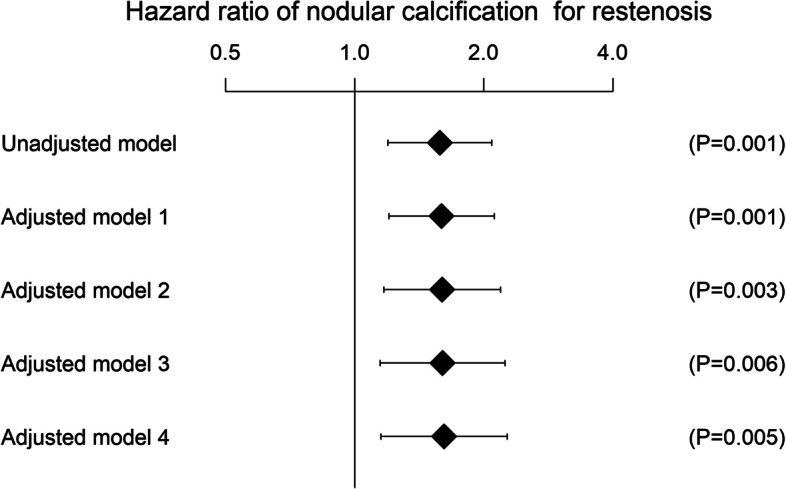
Association between the presence of nodular calcification and restenosis risk. Multivariate model 1 included nodular calcification and patient characteristics (sex, age, current smoking, diabetes mellitus, and renal failure on dialysis). Multivariable model 1 included nodular calcification, patient characteristics, and limb characteristics (chronic limb-threatening ischemia, history of revascularization, presence of popliteal lesions, reference vessel diameter, lesion length, chronic total occlusion, and PACSS classification). Multivariate model 3 included nodular calcification, patient characteristics, limb characteristics, and IVUS findings (calcification arc and distal EEM area). Multivariable model 4 included nodular calcification, patient characteristics, limb characteristics, IVUS findings, and procedural factors (IN.PACT Admiral use, minimum lumen area, and subintimal wire passage)

As shown in Fig. 3, the presence of nodular calcification was independently associated with restenosis risk; the adjusted hazard ratio was 1.62 (95% CI, 1.15 to 2.27; P = 0.005). From the interaction analysis, CLTI versus intermittent claudication did not have a significant interaction effect on the association between NC and clinical outcome (P for interaction = 0.90 for restenosis, 0.74 for TLR, and 0,25 for major amputation).

## Discussion

This study highlights the significant effect of NC on the clinical outcomes of femoropopliteal lesions treated with DCB angioplasty. The findings revealed that patients with NC had significantly lower 3-year primary patency rates than those without NC, demonstrating the adverse effects of NC on restenosis after DCB angioplasty.

The lower primary patency observed in the NC [+] group (53.8% vs. 65.8%, p = 0.001) was consistent with the hypothesis that NC contributes to suboptimal procedural outcomes. Furthermore, NC was independently associated with restenosis risk, with an adjusted hazard ratio of 1.61 (95% CI: 1.15 to 2.26, p = 0.006).

In addition, subgroup analysis in patients with CLTI showed no significant difference in clinical outcomes between the NC [+] and NC [-] groups, which may be due to the relatively small sample size. However, interaction analysis revealed no significant effect modification by clinical presentation (CLTI vs. intermittent claudication), suggesting that the adverse impact of NC on clinical outcomes was consistent across different clinical presentations.

NC is a significant challenge in EVT, particularly in DCB angioplasty. Specifically, it creates a rigid, uneven surface within the arterial wall, impairing vessel expansion and hindering drug delivery from the DCB. Consequently, the efficacy of DCB therapy may be compromised, negatively affecting clinical outcomes.

A potential mechanism contributing to restenosis in NC is the nonuniform distribution of antiproliferative agents (e.g., paclitaxel). Owing to the rigid and irregular nature of the NC, drug penetration into the arterial wall may be impeded, thereby reducing its effectiveness in inhibiting smooth muscle cell proliferation and neointimal hyperplasia.

Moreover, NC can alter local hemodynamics, exacerbate flow disturbances, and modify shear stress patterns, all of which may contribute to endothelial dysfunction and neointimal proliferation, thereby promoting restenosis. Additionally, the protrusive nature of NC may lead to the malapposition of devices, resulting in inadequate lesion coverage and an increased likelihood of long-term procedural failure.

Although these mechanisms remain theoretical, they align with those of previous studies demonstrating the adverse effects of severe calcification on drug delivery and vessel compliance [14]. Further investigations, including histopathological analyses and computational modeling, are necessary to validate these hypotheses and optimize the treatment strategies for NC-associated lesions.

Our findings underscore the importance of accurately identifying NC during pre-procedural planning. IVUS played a pivotal role in this study by enabling the precise detection and characterization of calcifications, which is critical for tailoring treatment strategies. Compared to conventional imaging modalities, such as angiography, IVUS offers superior resolution and allows for the assessment of calcification.

These findings raise important clinical considerations regarding the management of femoropopliteal lesions in NC. Current evidence suggests that adjunctive techniques, such as atherectomy or intravascular lithotripsy, may be beneficial for modifying calcifications prior to DCB therapy [15–17].

By facilitating better vessel compliance and optimizing drug absorption, these preparatory steps could improve the long-term outcomes in patients with NC. Additionally, alternative approaches such as DES, interwoven stents, or covered stents may be warranted for lesions in which NC severely limits DCB efficacy.

This retrospective, non-randomized design has several limitations, including potential selection bias and variability in device selection based on operator discretion. Additionally, the absence of atherectomy devices and lack of evaluation by an independent core laboratory may have influenced the outcomes. Despite these limitations, our findings provide valuable insights for NC management. However, further prospective randomized trials are required to validate these results and establish standardized protocols for the evaluation and treatment; further prospective, randomized trials are essential.

## Conclusion

NC is an independent predictor of restenosis in patients undergoing DCB angioplasty for femoropopliteal lesions. Patients with NC exhibit significantly lower primary patency rates, underscoring the need for advanced imaging modalities, such as IVUS, to guide treatment strategies. Optimizing vessel preparation using adjunctive techniques and exploring alternative therapeutic approaches may mitigate the adverse effects of NC on clinical outcomes. Further research is required to establish evidence-based guidelines for the management of calcified femoropopliteal lesions.

## Supplementary Information

Supplementary Material 1: Supplemental Figure. Kaplan–Meier estimates of freedom from restenosis (A), TLR (B), and limb salvage (C) in patients with CLTI. The dotted lines indicate the 95% confidence intervals. NC, nodular calcification; SE, standard error; TLR, target lesion revascularization; CLTI, chronic limb-threatening ischemia

## Data Availability

Due to the nature of this research, participants of this study did not agree for their data to be shared publicly, so supporting data is not available.
