# Supplementary figures and images for: Analysis of the impact of nodular calcification on clinical outcome after drug-coated balloon angioplasty for femoropopliteal lesions
Source: CVIR Endovasc. 2025 Aug 16;8:65. doi: 10.1186/s42155-025-00583-6 (PMC12357817; doi:10.1186/s42155-025-00583-6)

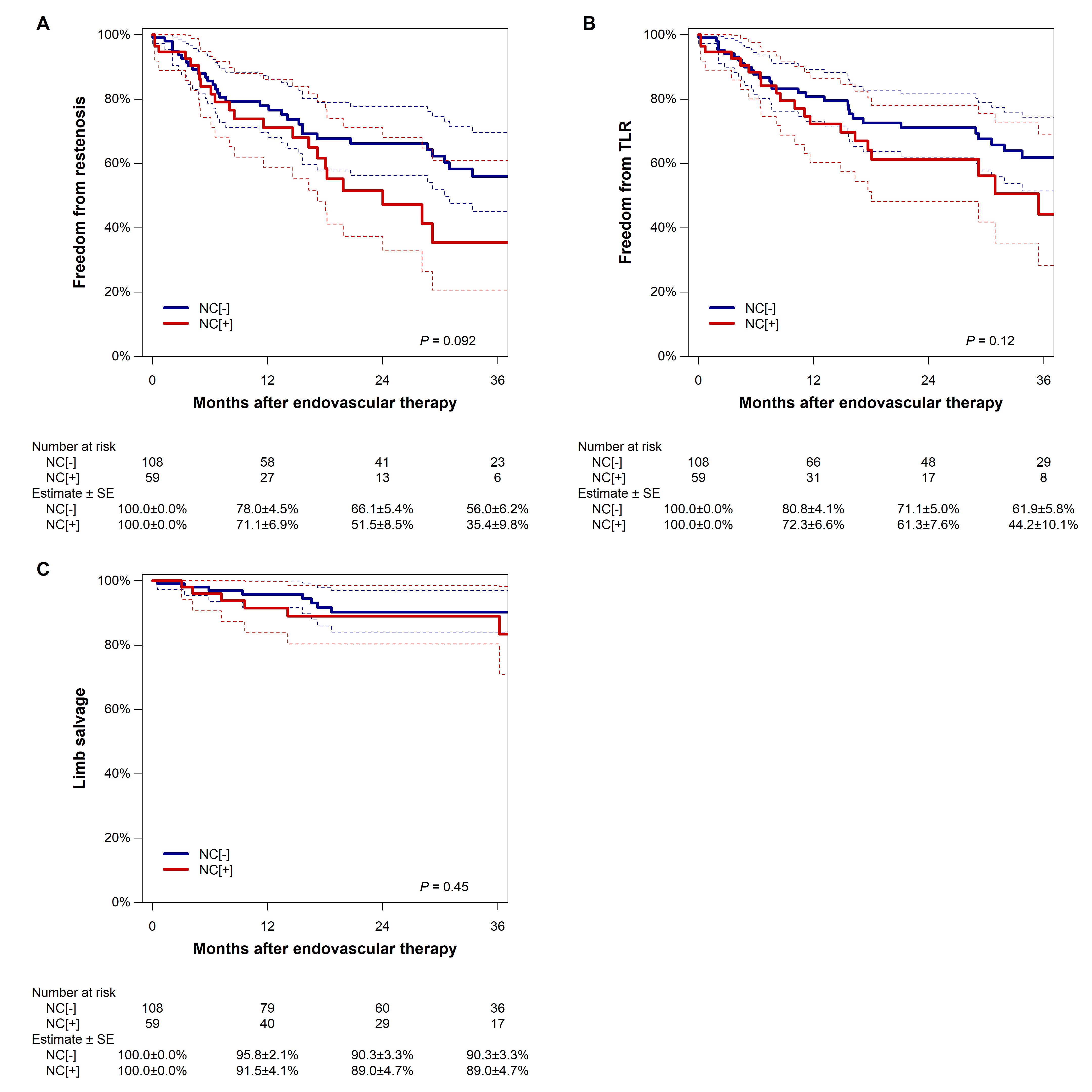

Supplement: Supplementary file 1 — Supplementary Material 1: Supplemental Figure. Kaplan–Meier estimates of freedom from restenosis (A), TLR (B), and limb salvage (C) in patients with CLTI. The dotted lines indicate the 95% confidence intervals. NC, nodular calcification; SE, standard error; TLR, target lesion revascularization; CLTI, chronic limb-threatening ischemia [file 42155_2025_583_MOESM1_ESM.tiff]
